# Screening of *Lactobacillus plantarum* subsp. *plantarum* with Potential Probiotic Activities for Inhibiting ETEC K88 in Weaned Piglets

**DOI:** 10.3390/molecules25194481

**Published:** 2020-09-29

**Authors:** Weiwei Wang, Hao Ma, Haojie Yu, Guangyong Qin, Zhongfang Tan, Yanping Wang, Huili Pang

**Affiliations:** 1 Henan Key Lab Ion Beam Bioengineering, School of Agricultural Sciences, Zhengzhou University, Zhengzhou 450052, China; wangwei508@foxmail.com (W.W.); mahaoworks@foxmail.com (H.M.); haojie-yu@foxmail.com (H.Y.); qinguangyong@zzu.edu.cn (G.Q.); tzhongfang@zzu.edu.cn (Z.T.); wyp@zzu.edu.cn (Y.W.); 2 School of Physics and Microelectronics, Zhengzhou University, Zhengzhou 450052, China

**Keywords:** *L. plantarum* subsp. *plantarum*, ETEC K88, antimicrobial, probiotics

## Abstract

For screening excellent lactic acid bacteria (LAB) strains to inhibit enterotoxigenic *Escherichia coli* (ETEC) K88, inhibitory activities of more than 1100 LAB strains isolated from different materials, and kept in the lab, were evaluated in this study. Nine strains with inhibition zones, at least 22.00 mm (including that of a hole puncher, 10.00 mm), and good physiological and biochemical characteristics identified by 16S DNA gene sequencing and *rec*A gene multiple detection, were assigned to *Lactobacillus* (*L.*) *plantarum* subsp. *plantarum* (5), *L. fermentum* (1), *L. reuteri* (1), *Weissella cibaria* (1) and *Enterococcus faecalis* (1), respectively. As investigated for their tolerance abilities and safety, only strain ZA3 possessed high hydrophobicity and auto-aggregation abilities, had high survival rate in low pH, bile salt environment, and gastrointestinal (GI) fluids, was sensitive to ampicillin, and resistant to norfloxacin and amikacin, without hemolytic activity, and did not carry antibiotic resistance genes, but exhibited broad spectrum activity against a wide range of microorganisms. Antibacterial substance may attribute to organic acids, especially lactic acid and acetic acid. The results indicated that the selected strain *L. plantarum* subsp. *plantarum* ZA3 could be considered a potential probiotic to inhibit ETEC K88 in weaned piglets for further research.

## 1. Introduction

Enterotoxigenic Escherichia coli (ETEC) K88 is a pathogenic variant of *Escherichia coli* that colonizes the surface of gastrointestinal cells when K88 infects the host and produces enterotoxins. This disrupts intestinal cellular electrolyte homeostasis, leading to fluid loss and, ultimately, to secretory diarrheal disease in newborns and piglets [1,2,3]. Post-weaning diarrhea (PWD) caused by ETEC K88 has a high mortality rate. In China and the United States, the average annual mortality of PWD cases is as high as 15% and 15.5%, respectively, and the direct economic losses amount to more than US $145 million [4]. Antibiotics have been long used to reduce diarrhea and enhance growth performance in weaned piglets, while long-term use of antibiotics caused the emergence of resistant strains of animal and food-borne pathogens and further intestinal microbial imbalance, according to announcement No. 194 of the Ministry of Agriculture and Rural Affairs. China officially stopped the production, import, operation, and use of some pharmaceutical feed additives as of 1 July 2020. Mineral compounds, i.e., zinc oxide (ZnO), have traditionally been used to mitigate post-weaning ETEC-F4 diarrhea [5,6]. Some studies have shown that organic acids, functional feedstuffs (blood plasma), feed enzymes, prebiotic oligosaccharides, and clay minerals can potentially prevent PWD associated with ETEC [7,8]. Due to environmental concerns, there are risks associated with microbial resistance, as well as difficulty of material preparation; thus, it is necessary to find a new effective alternative to antibiotics for the treatment and prevention of bacterial infection diseases, including diarrhea caused by ETEC K88.

It has been confirmed that probiotics are microorganisms that can have beneficial effects on host health, mainly through the production of antibacterial substances, competition with pathogenic bacteria for nutrients and adhesion sites, and enhancing intestinal mucosal barrier integrity to play an immunomodulatory role against diarrhea in weaned piglets [9]. Lactic acid bacteria (LAB) are the most frequently used probiotics, and several studies conducted on newly weaned piglets have noted that LAB can increase the abundance of lactobacilli and bifidobacteria, inhibit colonization of ETEC K88/F4, and improve production of short-chain fatty acid [10]. Patil et al. [11] found *Lactobacillus (L.) sobrius* may be effective in the reduction of *E. coli* F4 colonization, and may improve weight gain of infected piglets. Probiotic bacterium *L. rhamnosus* was effective in ameliorating PWD induced by *E. coli* K88, modulation of intestinal microflora, and enhancement of intestinal antibody defense [12]. In conclusion, the above research indicate that LAB have a good inhibitory effect on pathogenic bacteria in piglets, especially on intestinal imbalance caused by ETEC K88.

Excellent LAB strains should have characteristics of fast propagation, easy cultivation, strains to maintain a certain activity during production and storage, and a certain tolerance to processing technology in application, and the excellent probiotic LAB still need to be discovered. This study evaluates the probiotic properties of the LAB strains isolated from different sources by determining their inhibitory activities to ETEC K88. After determining physiological and biochemical characteristics, cell surface properties and tolerance to simulated human gastrointestinal (GI) tract and bile test, strains that performed very well were selected identified by 16S rRNA gene sequencing and recA gene multiple detection. Strains that performed well in the probiotic test were then evaluated for safety, and the antimicrobial substances and organic acids produced by the fermentation of the strains were determined.

## 2. Results

### 2.1. Inhibitory Activities to ETEC K88 of LAB Strains Isolated from Different Sources

Table 1 showed inhibitory activities to ETEC K88 of LAB strains isolated from different sources. As can be seen, 40 strains (ZA1 to ZA40, Zhengzhou University Agricultural College. Strains were uniformly rebranded as the ZA series for better documentation and preservation) with inhibition zones of at least 18.00 mm (including that of hole puncher diameter 10.00 mm, and the diameter of all inhibition zones below includes the diameter of the puncher) were selected for further study. Among these strains, ZA2, ZA3, ZA7, ZA8, ZA10, ZA15, ZA18, ZA19, ZA24, ZA28, ZA30, ZA33, and ZA34 had more than 22.00 mm inhibition zones. The remaining strains were eliminated from the primary screen because of poor inhibition, so they were not listed in the manuscript.

### 2.2. Physiological and Biochemical Characteristics of Selected LAB Isolates

Physiological and biochemical characteristics of selected LAB isolates are shown in Table 2. All strains were able to grow in 3.0 and 6.5 (*w*/*v*, %) NaCl, and at pH 4.0, 4.5, 5.0, 5.5, 6.0, 8.0, and 9.0. ZA1, ZA2, ZA3, ZA8, ZA9, ZA10, ZA11, ZA18, ZA28, ZA29, ZA33, ZA34, and ZA40 could grow at 5 °C, 50 °C, and pH 3.0, but other strains could not. In addition, ZA6, ZA15, ZA18, ZA26, ZA27, ZA31, ZA32, and ZA33 could produce gas from glucose, while other isolates could not.

### 2.3. 16S DNA Gene Sequence Analysis and recA Gene Multiple Detection

**Figure 1:**
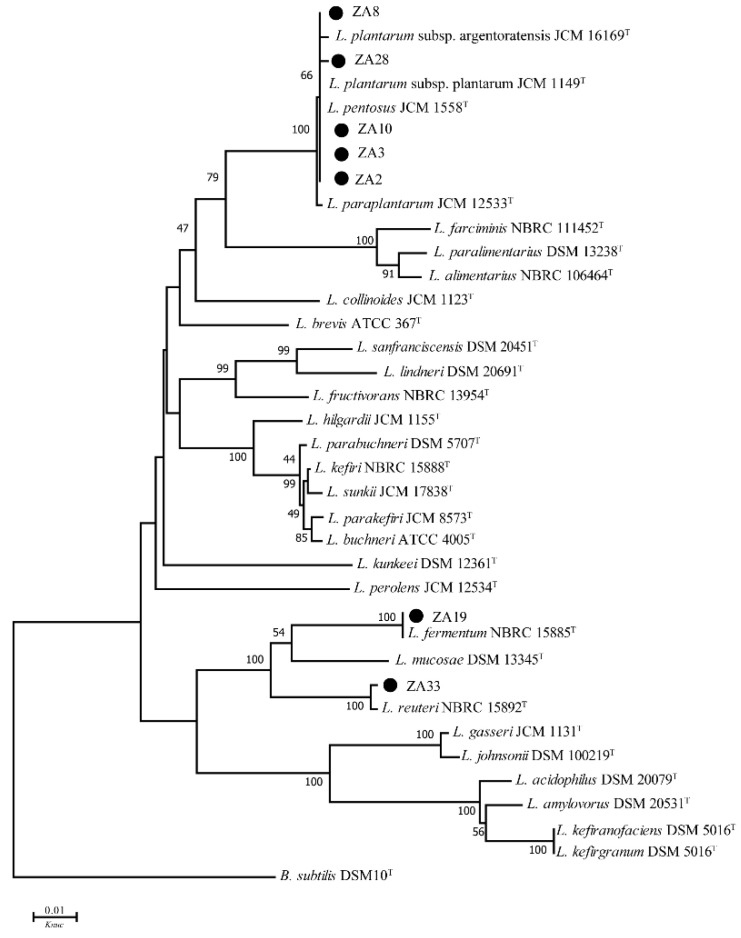
Phylogenetic tree of selected *Lactobacillus* strains. Bootstrap values for 1000 replicates are shown at the nodes of the tree, and *Bacillus subtilis* is used as an outgroup. The bar indicates 1% sequence divergence. *L.* = *Lactobacillus*, *B*. = *Bacillus*, *Knuc* = nucleotide substitution rate.

**Figure 2:**
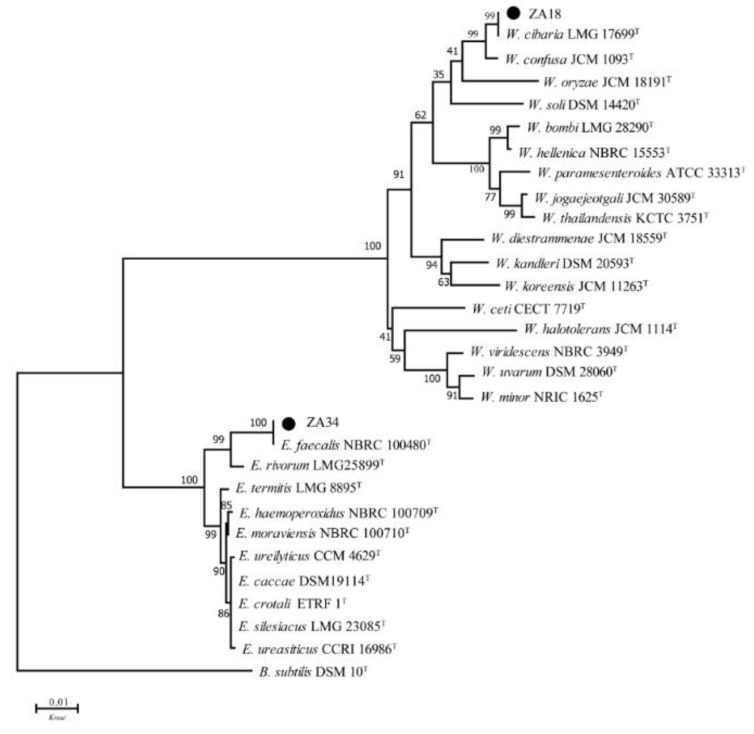
Phylogenetic tree of ZA18 and ZA34. Bootstrap values for 1000 replicates are shown at the nodes of the tree and *Bacillus subtilis* is used as an outgroup. The bar indicates 1% sequence divergence. *W.* = *Weissella*, *E.* = *Enterococcus*, *B*. = *Bacillus, Knuc* = nucleotide substitution rate.

Comprehensive inhibitory activity, physiological and biochemical results, ZA2, ZA3, ZA8, ZA10, ZA18, ZA19, ZA28, ZA33, and ZA34 were selected for 16S DNA gene analysis. The 16S DNA sequence analyzing was used for determining the molecular classification of representative LAB strains, and phylogenetic trees that were constructed using nine strains, based on evolutionary distances determined by a neighbor-joining method (shown in Figure 1 and Figure 2). These nine strains were placed in the cluster comprised of the genera *Lactobacillus*, *Weissella* (*W.*) and *Enterococcus* (*E*.). 

**Figure 3:**
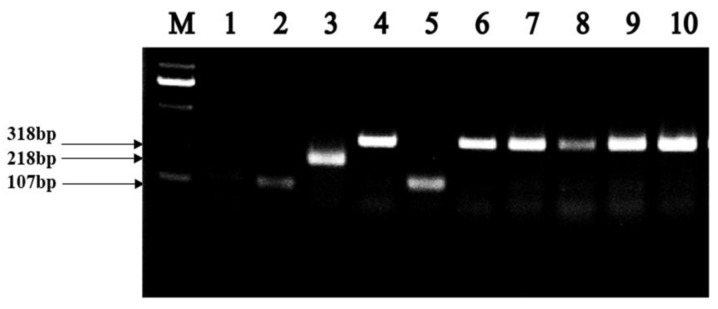
Amplification products obtained from the *recA* multiplex assay. Lane M contained a 2000 bp PLUS DNA ladder. Lanes 1, 2, 3, 4 and 5, PCR amplification products from *L. casei* JCM 16167^T^ (negative control), *L. paraplantarum* JCM 12533^T^, *L. pentosus* JCM 1558^T^, *L. plantarum* subsp. *plantarum* JCM 1149^T^, and *L. plantarum* subsp. *argentoratensis* JCM 16169^T^, respectively; lane 6, 7, 8, 9, and 10, PCR amplification product from ZA3, ZA2, ZA28, ZA8 and ZA10.

As shown in Figure 1, strains ZA2, ZA3, ZA8, ZA10, and ZA28 placed in the *L. plantarum* cluster, could not be distinguished by 16S DNA sequencing. The *rec*A gene PCR amplification products of these five strains, and the type strains in the *L. plantarum* cluster, including *L. casei*, *L. paraplantarum*, *L. pentosus*, *L. plantarum* subsp. *plantarum,* and *L. plantarum* subsp. *argentoratensis,* were shown in Figure 3. As can be seen, these five strains, and *L. plantarum* subsp. *plantarum* JCM 1149^T^, had 318 bp products, while the negative control *L. casei* did not produce any amplicons. Thus, strains ZA2, ZA3, ZA8, ZA10, and ZA28 could be assigned to *L. plantarum* subsp. *plantarum*, as ZA19 and ZA33 were also placed in the cluster of the genus *Lactobacillus*, and they could be identified as *L. fermentum* and *L. reuteri*-both supported by 100% bootstrap values, respectively, and both between 99% shared similarity in their 16S DNA.

In Figure 2, strain ZA18 was placed in the *Weissella* cluster, with the species *W. cibaria* LMG 17699^T^ being the most closely related species, which was supported by 99% bootstrap analysis in the phylogenetic tree and more than 99% similarity in the 16S rRNA gene sequence. Therefore, strain ZA18 belonged to *W. cibaria*. Strain ZA34, placed in the cluster of the genus *Enterococcus* in the phylogenetic tree, was clearly identified as *Enterococcus faecalis*, since it formed a very well-defined cluster (100% bootstrap) with *E. faecalis.*

### 2.4. Cell Surface Properties of Selected LAB Isolates

**Figure 4:**
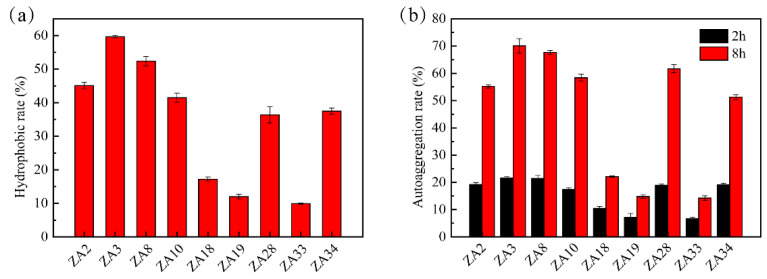
Cell surface hydrophobicity and auto-aggregation ability of lactic acid bacterial isolates. (**a**) hydrophobicity of selected LAB isolates; (**b**) auto-aggregation ability of selected LAB isolates.

The hydrophobicity and auto-aggregation ability of nine selected LAB isolates are shown in Figure 4. Figure 4a exhibited strains had significant differences in hydrophobicity, among these nine strains, ZA3 had the highest hydrophobicity at 59.7%, and the lowest was ZA33, only 9.9%. The auto-aggregation ability of isolates was presented in Figure 4b; all tested isolates showed lower auto-aggregation ability at 2 h, while at 8 h increased significantly. ZA3 also showed the highest auto-aggregation at 78.95% in comparison to other LAB isolates tested. Thus, ZA2, ZA3, ZA8, ZA10, ZA28, and ZA34 were used for further testing.

### 2.5. Acid Production Capacity and Growth Curve of Selected LAB Isolates 

**Figure 5:**
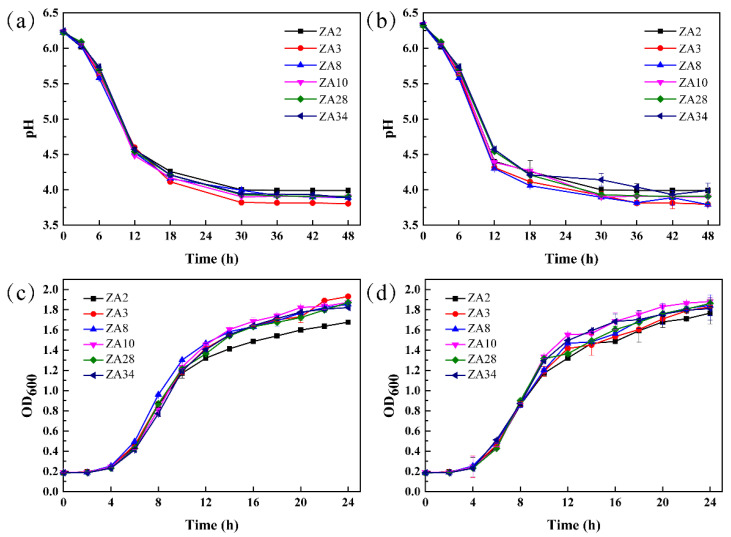
Acid production capacity and growth curve of six lactic acid bacterial isolates. (**a**,**b**), acid production curve of six LAB isolates cultivated at 30 and 39 °C, respectively; (**c**,**d**), growth curve of six LAB isolates cultivated at 30 and 39 °C, respectively.

Acid production capacity and growth curve of six selected LAB isolates cultivated at 30 and 39 °C are shown in Figure 5. There was no significant difference in the acid-producing ability of ZA2, ZA3, ZA8, ZA10, ZA28, and ZA34 within 48 h, among which ZA3 had the lowest pH at 3.0 after 48 h fermentation (Figure 5a,b), moreover, strains showed stronger acid production ability under cultivated at 39 °C compared with 30 °C. Figure 5c and d show the growth curve of these six isolates in 24 h cultivate at 30 and 39 °C, respectively, and from which one could see that the growth adaptation period of all six strains was from 0 to 2 h, and the logarithmic growth period was from 2 to 16 h.

### 2.6. pH 2.5 and Bile Salt Resistance of Selected LAB Isolates.

**Figure 6:**
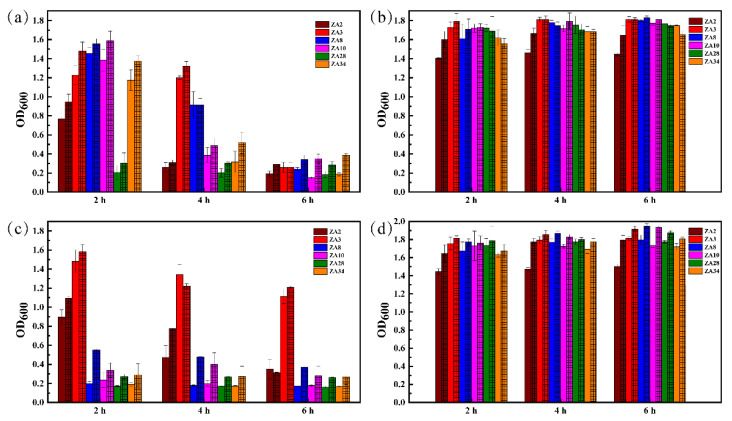
Biomass of six LAB isolates grown for 20 h after treatment in different acidic or bile salt environments with different times; (**a**) pH 2.5 treatment and cultivate at 30 and 39 °C, (**b**) pH 6.2 treatment and cultivate at 30 and 39 °C, (**c**) 0.5 % bile salt treatment and cultivate at 30 and 39 °C, and (**d**) 0 % bile salt treatment and cultivate at 30 and 39 °C.

Figure 6a and b separately showed pH 2.5 and pH 6.2 resistance of six selected LAB isolates cultivated at 30 and 39 °C. Optical density (OD) values of all six test LAB isolates were significantly different after being treated at pH 2.5 for 2, 4, and 6 h. All isolates had certain vigor after 2 h incubation at 30 °C, of which the highest was ZA8 with 1.456. After 4 h incubation at 30 °C, only ZA3 and ZA8 still had certain vigor, and ZA3 with 1.200 OD value. For 39 °C, all isolates had certain vigor after 2 h incubation, of which ZA3, ZA8, ZA10, and ZA34 with the OD value was 1.478, 1.566, 1.585, and 1.376, respectively. After 4 h incubation, ZA3 still had 1.322 OD value. As for 6 h incubation at 30 and 39 °C, no isolate had viability.

From Figure 6c, d, all six LAB isolates tested exhibited different viability after exposure to 0.5% bile salt and control for 2, 4, and 6 h. At 30 °C, only ZA2 and ZA3 had vitality with OD 0.896, and 1.481 after 2 h incubation, respectively, while other strains had no activity. After 4 h incubation, all strains were almost inactive (OD value of ZA2 was 0.474) except ZA3, which still had 1.341 OD value; for 6 h, the OD value of ZA3 was also 1.115. Regarding 39 °C, only ZA2 and ZA3 had vitality with OD 1.096 and 1.583 after 2 h incubation, while other strains had no activity; after 4 h incubation, only ZA3 had 1.219 OD value; after 6 h treatment, the OD value of ZA3 was also 1.210.

Based on the excellent pH 2.5 and bile salt tolerance of ZA3 in selected isolates, only ZA3 was selected for subsequent experiments.

### 2.7. Survival of ZA3 after Simulation Gastrointestinal (GI) Exposure 

**Figure 7:**
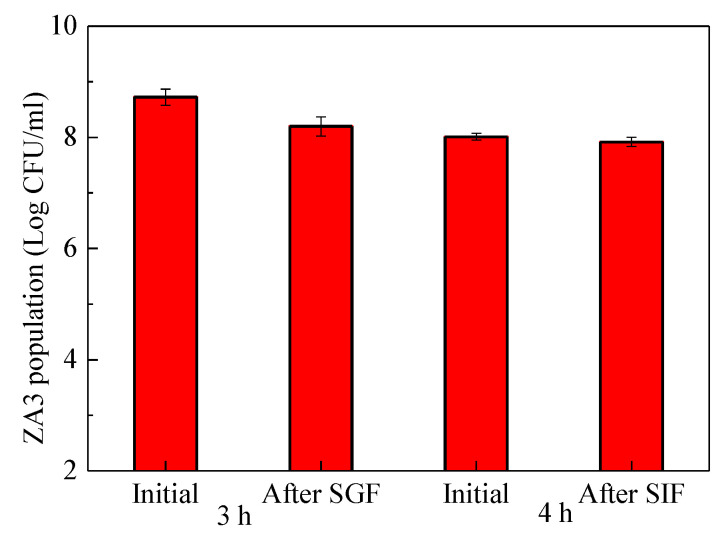
Surviving of ZA3 during a simulated gastrointestinal (GI) exposure.

Figure 7 illustrates the viable count (log colony forming units, CFU/mL) of the strain ZA3 during GI exposure. The population of ZA3 before in simulated gastric fluid (SGF) was 8.72 log CFU/mL, while after 3 h incubation in SGF, the population of ZA3 was 8.20 log CFU/mL, and the survival ratio was 94.03%. As for simulated intestinal fluid (SIF), an initial population of ZA3 was 8.01 log CFU/mL, the population reduced to 7.92 log CFU/mL after 4 h treatment, which was, to say, the survival ratio of ZA3 after the SIF phase was 98.88%.

### 2.8. Safety Evaluation of ZA3

#### 2.8.1. Safety Properties of ZA3

The results of virulence factor genes, biogenic amine genes, and antibiotic resistance gene amplification of ZA3 are shown in Table 3. PCR results revealed that ZA3 did not harbor any virulence genes, such as collagen genes associated with adhesin (ace), gelatinase gene (gelE), and the cytolysin gene (cylA), biogenic amine genes, such as histidine decarboxylase (hdc), tyrosine decarboxylase (tdc), and ornithine decarboxylase (odc), and antibiotic resistance genes, such as the vancomycin resistance gene (vanA) and the tetracycline resistance gene (tetM). 

#### 2.8.2. Assessment of Antibiotic Susceptibility

Table 4 shows antibiotic susceptibility of isolate *L. plantarum* subsp. *plantarum* ZA3, and the cut-off value for antibiotic resistance of LAB are also shown, which demonstrated the strain was sensitive to carbenicillin, ampicillin, erythromycin, chloramphenicol and amikacin, the minimum inhibitory concentration (MIC) were 0.5, 1, 1, 8 and 20 µg/mL, respectively, and resistant to cefamezin, gentamicin, norfloxacin, clindamycin and penicillin, the MIC were 10, 64, >512, 8 and 64 µg/mL, respectively.

#### 2.8.3. Hemolytic Activity of ZA3

**Figure 8:**
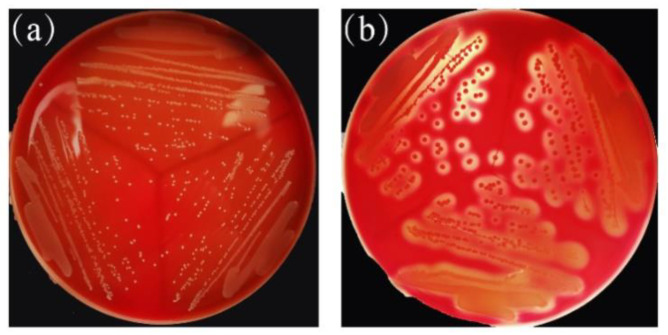
Hemolytic activity of ZA3; (**a**) ZA3, (**b**) positive control: *Staphylococcus aureus* ATCC 6538^T^.

As shown in Figure 8, compared with positive control, *Staphylococcus aureus* ATCC 6538^T^, having blood hemolysis activity in Figure 8b, ZA3 in Figure 8a showed no hemolytic activity.

#### 2.8.4. Toxicity Test of the ZA3 Strain against intestinal epithelial (IPEC-J2) Cells

**Figure 9:**
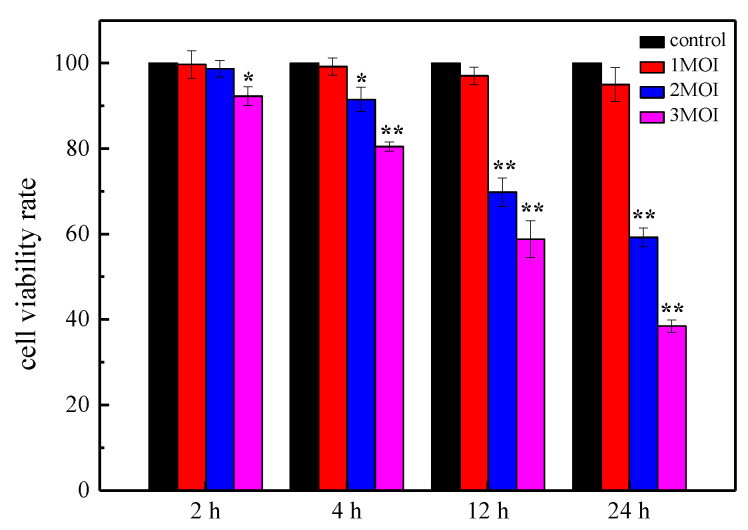
Cell viability rates of intestinal epithelial (IPEC-J2) after different time incubation with different concentrations of ZA3. The asterisk denotes a significant difference compared with control (* p < 0.05, ** p < 0.01).

Cell Counting Kit-8 (CCK-8) results are shown in Figure 9. When treated with 1 multiplicity of infection (MOI ZA3), the cells showed normal activity at all time periods, which was not significant compared to the control (*p >* 0.05). As for 2 MOI ZA3, after 2 h reaction, cell viability was slightly reduced, and it did not damage cells or affect cell viability after 4 h reaction. Regarding being treated with 3 MOI ZA3, cells were severely damaged, and the survival rate was significantly reduced compared with control cells (*p <* 0.05). After 4 h reaction, the cell survival rate was significantly reduced (*p <* 0.01). Moreover, cell survival rate was significantly reduced, and cells were severely damaged (*p <* 0.01) after being treated with 2 and 3 MOI for 12 and 24 h.

### 2.9. Antimicrobial Spectrum Test of ZA3

Results of the agar well diffusion in Table 5 displayed that ZA3 demonstrated powerful antimicrobial activity against all selected microorganisms tested in this work. As can be seen, inhibition zone diameters of more than 22.00 mm were *Pseudomonas aeruginosa* ATCC 15692^T^ and *Listeria monocytogenes* ATCC 51719^T^, as for *Escherichia coli* ATCC 11775^T^, *Staphylococcus aureus* ATCC 6538^T^, *Bacillus subtilis* ATCC 19217^T^, *Micrococcus luteus* ATCC 4698^T^, and *Salmonella enterica* ATCC 43971^T^, inhibition zone diameters were all above 18.00 mm.

### 2.10. Carbohydrate Utilization Patterns of ZA3

Carbohydrate utilization patterns of ZA3 are shown in Table 6, and results indicate that ZA3 could use galactose, D-glucose, D-fructose, mannose, L-sorbose, rhamnose, mannitol, sorbitol, maltose, lactose, melibiose, saccharose, trehalose, gluconate, N-acetyl glucosamine, and amygdalin as carbon sources, and ribose could be weakly used. Other than that, the remaining carbon sources, such as glycerol, D-arabinose, L-arabinose, D-xylose, dulcitol, inositol, salicin, melezitose, D-raffinose, starch, xylitol, and L-arabitol were completely unavailable.

### 2.11. Identification of the Antimicrobial Substance Produced by ZA3

Effects of pH, enzymes, and hydrogen peroxide on the antimicrobial activity of ZA3 against ETEC K88 are shown in Table 7. The inhibition zone diameter of fermentation liquid and supernatant of ZA3 were both above 18.00 mm. The results summarized that the hydrogen peroxide did not affect antimicrobial activity of ZA3 based on the inhibition zone diameter, and were still above 18.00 mm after being treated by hydrogen peroxide. At the same time, after being treated with pepsinum and tryptase, the inhibition zone diameter was still above 18.00 mm. As for proteinase *K*, the diameter was also between 14.00–18.00 mm. However, when tested the effects of different pH, the antimicrobial activity of ZA3 decreased with increasing pH, which was not affected by pH 3.0 and 4.0, obviously, but slightly decreased at pH 4.5. A complete loss of activity was observed at pH values ranging from 5.5 to 10.0. Therefore, antibacterial substances produced by ZA3 may be acid.

### 2.12. Organic Acid Produced by Fermentation of ZA3

**Figure 10:**
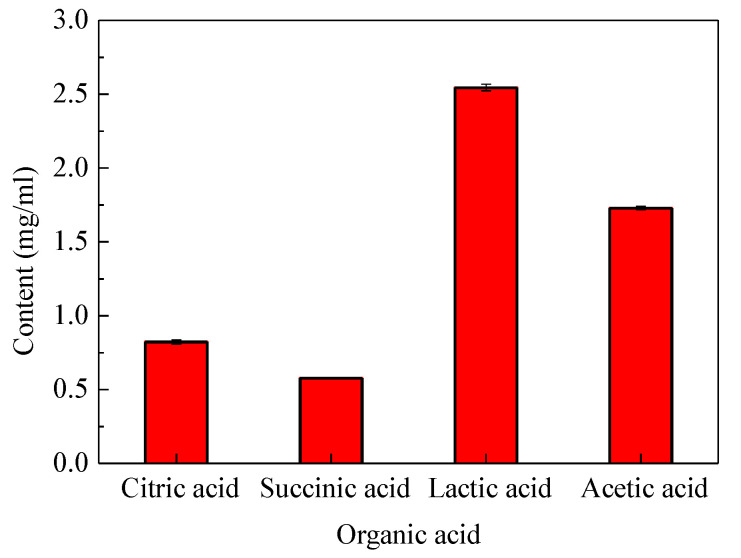
The organic acid produced by fermentation of ZA3.

Liquid chromatography was used to analyze the acid production of ZA3 after 24 h fermentation. As shown in Figure 10, four kinds of organic acid were mainly detected in the fermentation broth: citric acid, succinic acid, lactic acid, and acetic acid. The content was 0.822, 0.576, 2.545, and 1.729 mg/mL, respectively.

## 3. Discussion

In this study, to obtain functional probiotic bacteria that inhibit ETEC K88, more than 1100 LAB from different sources were tested and 40 isolates with an inhibition zone diameter of 18.00 cm or more were selected. ETEC is the main pathogenic bacterium that causes diarrhea in humans and young animals. As one of the indispensable floras with important physiological functions in human and animal bodies, LAB has the incomparable host bacteria advantages of other receptor strains. Sirichokchatchawan et al. [13] reported the strain *L. plantarum* showed antagonistic activity against *E. coli;* a 6.0–9.00 mm inhibitor zone diameter was considered to be weak inhibition and marked as +. Moreover, 10.0–13.00 mm (intermediate inhibition) was marked as ++, 14.0–16.00 mm (strong inhibition) was marked as +++, and more than 17.00 mm (very strong) was marked as ++++ by the agar well diffusion method (including that of the hole puncher, 6.00 mm). While in this study, 13 isolates had more than 22.00 mm inhibition zones by using the same test method, the only difference being that the diameter of the hole puncher used was 10.00 mm.

One of the most important criteria for selecting LAB with probiotic properties is the ability to tolerate the environment of the gastrointestinal tract, with the pH of pig gastric juices being as low as 2.0, and bile with a pH of about 8.0. Dushku et al. [14] isolated and identified 40 LAB strains from the gastrointestinal tract of local snails, which were artificially simulated to survive the gastrointestinal tract of only two strains. Rao et al. [15] screened *L. plantarum* AT4 for low pH and up to 0.5% bile salts. This study found 40 selected strains with excellent inhibition abilities; 13 homo-fermentative isolates could grow at 5 to 50 °C, and pH 3.0–10.0, and ferment glucose without producing gas. Considering that the temperature of weaned piglets is usually around 38.5 to 40 °C, the study examined acid production capacity, growth situation, low pH, and bile salt tolerance of selected LAB strains at 39 °C in addition to the optimal 30 °C conditions. As can be seen, all six selected strains showed stronger acid production ability under 39 °C, compared with 30 °C, and the growth of strains were not affected at 39 °C. Additionally, there was no significant difference between pH 2.5 and 0.5% bile salt resistance of the six selected LAB strains cultured at 30 and 39 °C. These results further suggest that the selected strains have potential for application as animal intestinal probiotics. Relative to heterofermentative LAB, producing a mix of lactic acid and acetic acid, homofermentative LAB promotes rapid fermentation, primarily producing lactic acid and rapidly reducing the pH, preventing the growth of other undesirable spoilage organisms, which indicated that these strains satisfied the demands for growth in relatively extreme environments. In a word, the excellent physiological and biochemical characteristics provide tremendous potential for selected strains in practical applications [16].

Species identification is the basis for conducting scientific experiments, and the identification of the genus provides an indirect understanding of the bacteria’s habits, metabolism, and pathogenic patterns. In this study, phylogenetic trees were constructed based on the evolutionary distances of their 16S DNA sequences using the neighbor-joining method to identify selected LAB isolates at the species level. Selected strains were placed in clusters comprised of genera *Lactobacillus*, *Weissella,* and *Enterococcus*, since strains ZA2, ZA3, ZA8, ZA10, and ZA28 were clearly assigned to the genus *Lactobacillus,* and grouped in the *L. plantarum* cluster on the phylogenetic tree with type strains *L. casei* JCM 16167^T^, *L. paraplantarum* JCM 12533^T^, *L. pentosus* JCM 1558^T^, *L. plantarum* subsp. *plantarum* JCM 1149^T^, and *L. plantarum* subsp. *argentoratensis* JCM 16169^T^, which could not be separated by 16S rDNA [17]. By means of *rec*A gene, PCR amplification products, these five strains and *L. plantarum* subsp. *plantarum* JCM 1149^T^ had 318 bp products, while the negative control *L. casei* and the other three type strains did not produce any amplicons; thus, ZA2, ZA3, ZA8, ZA10, and ZA28 strains could also be accurately identified as *L. plantarum* subsp. *plantarum*.

Adherence is a prerequisite for strains to colonize the gut and increase their viability. Only if the strain is able to adhere and colonize the intestinal tract can it promote immune regulation and stimulate the intestinal barrier and metabolism function [18]. Adherence mainly includes cell surface hydrophobicity, self-aggregation, and co-aggregation to indicator bacteria, and its effect on intestinal cells. It has been shown that high hydrophobicity and auto-aggregation ability could promote the colonization of beneficial microorganisms in the gastrointestinal tracts of human hosts [19]. In this study, ZA3 from weaned pig feces showed the highest hydrophobic activity (59.70%) and auto-aggregation ability (78.95%) in comparison to other LAB isolates tested, and the results are consistent with previous studies that the higher the surface hydrophobicity of these isolates, the stronger the self-aggregation ability [20]. 

As a functional LAB, it should overcome several challenging environmental conditions, such as extremely high or low pH, salt, and bile, all are the most common factors [21]. In the present study, it was found that ZA3 still had certain vigor after 4 h incubation in different acidic or bile salt environments, and exhibited 94.03% survival rate after 3 h in simulated gastric juice. The results obtained by Joghataei et al. [22] displayed that *L. fermentum* FH19 exhibited the highest survival rate (96%) after 3 h in SGF. Comparatively speaking, ZA3 had 98.88% survival ratio after the SIF phase in this study, indicating that it has a significant effect on SGF and SIF tolerance. Lee et al. [23] reported *L. plantarum* C182 possessed a significant level of resistance against 0.3% bile salts, while ZA 3 had vitality with OD value 1.115 after exposure to 0.5% bile salt for 6 h. 

Safety is also one of the basic criteria for screening strains, LAB, especially *Lactobacillus*, are usually considered safe. However, it has recently been discovered that *Lactobacillus* and *Bifidobacterium* are frequently isolated from diseased tissues, such as endocarditis and sepsis [24,25]. Therefore, it is necessary to re-evaluate their safety. In addition, the safety evaluation of probiotics has become more important with the isolation and application of new probiotics and the emergence of genetically modified probiotics. As isolated strains *Lactobacillus* MMP4 wanted to be used in the dairy industry, Choudhary et al. [26] used PCR to investigate whether strain MMP4 was an antibiotic resistant gene, and had safety parameters, such as gelatinase and hemolytic activity. In this study, all tested strains were PCR-negative for all the virulence factors, including virulence factor genes, biogenic amines genes, and antibiotic resistances genes, and antibiotic susceptibility and hemolytic activity, which are at risk for genetic transfer. The MIC of ZA3 to carbenicillin, cefamezin, ampicillin, clindamycin, erythromycin, and chloramphenicol were 0.5, 10, 1, 8, 1, and 8 µg/mL, which was consistent with the study by Nawaz et al. [27], respectively. CCK-8 assay for the cytotoxicity of ZA3 on IPJC-2 cells showed that the strain was not cytotoxic. In addition, there was absence of hemolysis on blood agar, indicating that ZA3 could have potential as a safety probiotic candidate.

The ultimate goal of screening LAB is application, and the ability to utilize a wider variety of carbon sources, indicating that the strain ZA3 was easy to culture and has greater viability, which is of greater value in research and production.

There is a wide variety of antibiotics, and antibiotic inhibition spectrum varies by antibiotic type, when using probiotics instead of antibiotics, which probiotics should be used instead of antibiotics? Is it a complete replacement or a partial replacement? It is difficult for feed and breeding companies to make these decisions. Moreover, probiotics typically exhibit narrow killing spectrum, inhibiting only those bacteria that are closely related to them. Therefore, it is very important to screen strains with a wide antimicrobial spectrum and define the range of antimicrobial spectrum of the strains. In this study, results of screening for the potential antagonistic activity against important pathogens showed ZA3 exhibit broad-spectrum activity against a wide range of microorganisms, including gram-positive and gram-negative bacteria. Additionally, because the intestinal flora is a complex system and it is difficult to introduce new microorganisms into this competitive environment, it produces substances, such as bacteriocins, organic acids, short-chain fatty acids, and hydrogen peroxide that inhibit pathogens in the gut of growth, and reduce the occurrence of diarrhea [28]. ZA3 had strong antimicrobial activity after excluding the effects of hydrogen peroxide by proteinase *K* and eliminating hydrogen peroxide from the cell-free culture supernatants; however, all activities disappeared completely when the cell-free supernatant was treated with neutralizing pH, which indicated that the antimicrobial activity may be attributed to the production of organic acids. Similarly, as one of the antimicrobial compounds, Tirloni et al. [29] and Bah et al. [30] proposed organic acids can significantly inhibit the growth of pathogenic bacteria. Silva et al. [31] reported *L. lactis* subsp. *lactis* presented antimicrobial activity against pathogens, which may be related to their lactic acid production, low pH values, and antimicrobial compounds. Furthermore, some research results showed that the main organic acids metabolites of LAB fermentation were pyruvate, lactic acid, acetic acid, citric acid, oxalic acid, and malic acid etc. [29,32,33]. ZA3 mainly produced four kinds of organic acids: citric acid, succinic acid, lactic acid, and acetic acid, after 24 h fermentation, respectively. Lactic acid and acetic acid were significantly higher than other two acids. Such a conclusion is the same as that by Mun et al. [34], who researched lactic acid and acetic acid in *L. plantarum* fermentation broth as the main antibacterial active substances. In summary, it is worthwhile to investigate ZA3’s effect on diarrhea in animals induced by ETEC K88.

## 4. Materials and Methods 

### 4.1. Screening of LAB Restrain ETEC K88 Activity 

More than 1100 LAB strains isolated from weaned piglet feces, feed grass, Qula, rice silage, mixture silage, and corn silage were used in this study. All LAB isolates were incubated in de Man Rogosa Sharpe (MRS) broth at 30 °C. 

Bacterial strains used in this article are shown in Table 8. Among which, ETEC K88 *Salmonella enterica* ATCC 43971^T^ and *Enterococcus faecalis* ATCC 29212^T^ were purchased from the China Veterinary Culture Collection Center (CVCC), China General Microbiological Culture Collection Center (CGMCC), and China Center of Industrial Culture Collection (CICC), respectively. *Staphylococcus aureus* ATCC 6538^T^, *Bacillus subtilis* ATCC 19217^T^, *Escherichia coli* ATCC 11775^T^, *Listeria monocytogenes* ATCC 51719^T^, *Pseudomonas aeruginosa* ATCC 15692^T^, and *Micrococcus luteus* ATCC 4698^T^ were stored in the laboratory. 

The antagonistic effect of LAB isolates against ETEC K88 were first determined by the agar well diffusion technique [35]. The target bacteria ETEC K88 was grown in Luria–Bertani (LB) liquid culture medium and incubated at 37 °C with 180 rpm for 12 h. Subsequently, 100 uL of the overnight culture of K88 was added to LB agar medium, cooled to 50 °C, and shaken, mixing well before pouring onto the surface of the already coagulated LB agar plates. After solidification, 200 μL 16 h cultures of different strains were placed in the hole punched in the center of the plate by using a hole puncher (diameter 10.00 mm), with uninoculated MRS broth as negative, and penicillin (pc) as a positive control, respectively. Inhibition zone was measured after 2 h diffusion at 4 °C.

### 4.2. Molecular Identification of Representative Strains

#### 4.2.1. 16S rRNA Analysis 

The 16S rRNA gene of selected strains were amplified by PCR using the 27 F (5′-AGAGTTTGATCCTGGCTCAG-3′) and 1492 R (5′-GGTTACCTTGTTACGACTT-3′) universal primer sets. Amplifications by PCR were performed in a total volume of 50 μL DNA thermal cycler, which contained 25 μL 2 × Taq Plus Mastermix, 27 F and 1492 R primer 1 μL each, and finally add sterile distilled water and fix to 50 μL. Single colonies cultured for 24 h were added to each reaction system, separately, and the PCR condition was: initial activation at 94 °C for 5 min; 33 cycles at 94 °C for 50 s, 52 °C for 50 s and 72 °C for 50 s; and a final cycle at 72 °C for 17 min. The PCR products were placed in a 1% agarose gel and electrophoresed with Ethidium Bromide (EB) solution staining. The successful amplification was analyzed by sequencing service (MGI Tech Co., Ltd., Beijing, China), and resulting sequences were compared with sequences in the GenBank database using the BLAST program available on the National Center for Biotechnology Information website (http://www.ncbi.nlm.nih.gov). 

#### 4.2.2. RecA Multiple Sequence

Means of partial amplification product comparison of the *rec*A gene was used to distinguish strains of *L. plantarum* cluster, including *L. casei*, *L. paraplantarum*, *L. pentosus*, *L. plantarum* subsp. *plantarum,* and *L. plantarum* subsp. *argentoratensis* [17]. A multiplex PCR assay (20 μL) was performed with *rec*A gene-based primers para F (5′-GTCACAGGCATTACGAA AAC-3′), pent F (5′-CAGTGGCGCGGTTGATATC-3′), plan F (5′-CCGTTTATGCGGAACACC TA-3′), and pREV (5′-TCGGGATTACCAAACATCAC-3′). The PCR mixture was composed of primers para F, pent F, and pREV (0.25 μM each), 0.12 μM primer plan F, 25 μL 2 × Taq Plus Mastermix, add sterile distilled water to 40 μL. Single colonies including type strains *L. casei* JCM 16167^T^ (negative control), *L. paraplantarum* JCM 12533^T^, *L. pentosus* JCM 1558^T^, *L. plantarum* subsp. *plantarum* JCM 1149^T^, and *L. plantarum* subsp. *argentoratensis* JCM 16169^T^, and strains of this cluster, indistinguishable after 16S rRNA analysis, were added to each reaction system, separately. The PCR condition was initial denaturation at 94 °C for 5 min, 33 cycles of denaturation at 94 °C (30 s), annealing at 56 °C (10 s), elongation at 72 °C (30 s), and final extension at 72 °C for 5 min. The PCR products were visualized in a 2% agarose gel in 0.5 × TAE (20 mM Tris-acetate, 0.5 mM EDTA, pH 8.0) buffer. 

### 4.3. Cell Surface Hydrophobicity and Auto-aggregation of Representative Strains

The cell surface hydrophobicity and auto-aggregation assay were performed according to Somashekaraiah et al. [20] and Wang et al. [36], respectively, and both with some modifications. LAB strains cultivated in MRS broth at 30 °C for 16 h were washed twice with phosphate-buffered saline (PBS) (8000× *g*, 4 °C, 10 min) and resuspended in PBS buffer followed by absorbance measurement at 600 nm (OD 600, marked as A0). 

For cell surface hydrophobicity analysis, 3 mL cell suspension was blended with 1 mL xylene; the two-phase system was mixed by vortexing for 2 min, and incubated at 37 °C without shaking for 30 min (for separation of the aqueous and organic phases). The water phase was carefully removed and its absorbance at 600 nm was measured (OD 600, marked as A1). The percentage of cell surface hydrophobicity (H%) was calculated using the following formula:

‘H% = (1 − A1/ A0) × 100% (1)

As for auto-aggregation, bacterial cell suspensions were mixed by vortexing for 10 s and incubated at room temperature for 8 h, the upper suspension was checked for absorbance at 600 nm at time intervals of 0, 2, and 8 h (OD 600, the absorbance at each particular time marked as different Atime). The auto-aggregation was measured (in percentage) using the following formula:

auto-aggregation% = [1 − (Atime/A0) × 100] % (2)

### 4.4. Determination of Growth Curve and Acid Production Capacity of Representative Strains

Each single LAB colony was picked and added into 20 mL sterile MRS, and the optical density at 600 nm (OD 600) and colony forming units (CFU) /mL were determined immediately at 2, 4, 6, 8, 10, 12, 14, 16, 18, 20, 22, and 24 h after inoculation at 30 and 39 °C, respectively. The pH of each fermentation solution was determined at 0, 6, 12, 18, 24, 30, 36, 42, and 48 h after inoculation at 30 °C and 39 °C, respectively. 

### 4.5. Low pH and Bile Salt Tolerance of Representative Strains

MRS broth at pH 2.5 and MRS solution containing 0.5% (*wt/vol*) bile salt were used to assess low pH and bile salt tolerance, and pH 6.8 and no bile salt MRS broth were set as control, respectively. Each LAB isolate was incubated at 30 °C and 39 °C in different acidic and bile salt environments for 0, 2, 4, and 6 h, respectively, and then incubated at 30 °C for 20 h, measuring the biomass by optical density at 600 nm. 

### 4.6. Survival of Representative Strains in GI Fluids

The simulated gastric fluid (SGF) and simulated intestinal fluid (SIF) were performed as described by Massounga et al. [37] with modifications. Briefly, for SGF, 3.5 g/L pepsin was suspended in 0.2% w/v sterile NaCl solution and adjusted pH to 2.0, made the total volume of the solution up to 100 mL, and filtered through a 0.22 μm filter membrane. For SIF, 1 g/L trypsin, 18 g/L bile salt from ox and 11 g/L NaHCO_3_ were suspended in 0.2% w/v sterile NaCl solution, adjusted the pH of the solution 6.8, and, again, brought the total solution volume to 100 mL with 0.22 μm membrane filtration. Moreover, 2% 10^8^ CFU/mL LAB solution was added to 20 mL SGF and incubated for 3 h (SIF was 4 h). Viable colonies were determined with plate counts on MRS agar after 0, 3, and 7 h incubation.

### 4.7. Pathogenicity Evaluation of Strain ZA3

#### 4.7.1. Safety Evaluation

The strain ZA3 was screened for the presence of genetic traits related to virulence factors, biogenic amines, and antibiotic resistance using the PCR protocols [38], and referring to the approach of Shankar et al. [39]. *Enterococcus faecalis* ATCC 29212^T^ that harbor the target virulence genes (ace, cylA, and gelE) was used as positive control, and Milli-Q water as positive control, respectively. The target genes include gelE (gelatinase) (F: 5′-TATGACAATGCTTTTTGGGAT-3′, R: 5′-AGATGCACCCG AAATAATATA-3′), cylA (cytolysin) (F: 5′-GAATTGAGCAAAAGTTCAATCG-3′, R: 5′-GTCTGTCTT TTCACTTGTTTC-3′), ace (adhesion of collagen) (F: 5′-ACTCGGGGATTGATAGGC-3′, R: 5′-GCTGC TAAAGCTGCGCTT-3′), vanA (vancomycin resistance) (F: 5′-TCTGCAATA GAGATAGCCGC-3′, R: 5′-GGAGTAGCTATCCCAGCATT-3′), tetM (tetracycline resistance) (F: 5′-ATTACACTTCCGATTT CGG-3′, R: 5′-GTTAAATAGTGTTCTTGGAG-3′), hdc (histidine decarboxylase) (F: 5′-AGATGGTAT TGTTTCTTATG-3′, R: 5′-AGACCATACACCATAACCTT-3′), tdc (tyrosine decarboxylase) (F: 5′-GA YATNATNGGNATNGGNYTNGAYCARG-3′, R: 5′-CCRTARTCNGGNATAGCRAARTCNGTRTG-3′), and odc (ornithine decarboxylase) (F: 5′-GTNTTYAAYG CNGAYAARCANTAYTTYGT-3′, R: 5′-ATNGARTTNAGTTCRCAYTTYTCNG GG-3′).

#### 4.7.2. Antibiotic Susceptibility

The minimum inhibitory concentration (MIC, µg/mL) of 10 antibiotics was determined by agar dilution method according to the Clinical and Laboratory Standards Institute (CLSI) standards. Each experiment was performed on MRS agar plates containing different antibiotics, including carbenicillin, cefamezin, ampicillin, gentamicin, norfloxacin, clindamycin, penicillin, erythromycin, chloramphenicol, and amikacin. Antibiotics were prepared at a range of concentrations from 0.125 to 512 µg/mL by twofold dilution, and each concentration was tested in triplicate. All isolates were inoculated on MRS agar containing antibiotics at 37 °C for 24 h, and strains cultivated on MRS agar plates without antibiotics were used as controls.

#### 4.7.3. Hemolytic Activity

Hemolytic activity was analyzed on blood agar as the manufacturer’s instructions. Fresh bacterial ZA3 was streaked on Columbia blood agar plates, and *Staphylococcus aureus* ATCC6538^T^ was used as positive control. 

#### 4.7.4. Toxicity Test of the ZA3 Strain against Eukaryotic Cells

Cell viability was determined by the Cell Counting Kit-8 (CCK-8) assay (Nanjing Jiancheng Institute of Bioengineering, Nanjing, China). The experiment was divided into normal cell and LAB treatment groups, and the multiplicity of infection (MOI) was set to three gradients, 1, 2, and 3, with each well performed in sextuplicate. IPEC-J2 cells (1 × 10^5^ cells/well) were seeded into 96-well tissue culture plates with 100 μL per well. After cells grown in a humidified chamber at 37 °C under 5% CO_2_, collected that it reached 80% confluence, CCK-8 was used to stain the cells for about 2, 4, 12, and 24 h after being treated by ZA3, respectively. The optical density was measured at 450 nm using a microplate spectrophotometer.

Cell viability rate = ((As − Ab) / (Ac − Ab)) × 100% (3)

As: experimental well (including cell-containing medium, CCK-8 and ZA3);

Ac: control well (including cell-containing medium and CCK-8);

Ab: blank well (cell culture medium without cells, CCK-8).

### 4.8. Antimicrobial Activity

The antimicrobial activity of ZA3 against pathogenic bacteria was assessed by the method of agar well diffusion, *E. coli* ATCC 11775^T^, *Pseudomonas aeruginosa* ATCC 15692^T^, *Staphylococcus aureus* ATCC6538^T^, *Bacillus subtilis* ATCC 19217^T^, *Listeria monocytogenes* ATCC 51719^T^, *Micrococcus luteus* ATCC 4698^T^*,* and *Salmonella enterica* ATCC 43971^T^ were used as indicator bacterium. 

### 4.9. Carbohydrate Utilization Patterns of ZA3

Thirty common carbon sources, including glycerol, D-arabinose, L-arabinose, ribose, D-xylose, galactose, D-glucose, D-fructose, mannose, L-sorbose, rhamnose, dulcitol, inositol, mannitol, sorbitol, salicin, cellobiose, maltose, lactose, melibiose, saccharose, trehalose, melezitose, D-raffinose, starch, xylitol, L-arabitol, gluconate, N-acetyl glucosamine, and amygdalin were used to detect carbohydrate utilization patterns of ZA3 by replacing the carbon source, each in turn, in the MRS medium.

### 4.10. Identification of the Antimicrobial Substance of ZA3

The antimicrobial substance of ZA3 was assessed according to Ni et al. [40] and exclusion experiments for acid and catalase inhibition, and protease (proteinase K, trypsin, and pepsin) degradation were performed, respectively. For the pH stability assay, the ZA3 bacteria suspension was adjusted to pH values ranging from 3.0 to 10.0 by 0.2 M HCl or 0.2 M NaOH. For the hydrogen peroxide, the ZA3 bacteria suspension was treated with 0.5 mg/ mL catalase to confirm if it had catalase inhibition. The effects of protease on antimicrobial activity were determined by incubating the ZA3 suspension, separately, with 1 mg/mL proteinase K, trypsin, and pepsin at 37 °C for 2 h, and collecting the supernatant of overnight LAB cultures in MRS broth by centrifugation at 8000× *g* for 10 min to assay.

### 4.11. Determination of Organic Acids Produced by ZA3 

The organic acid produced by ZA3 was determined with high performance liquid chromatography (HPLC) (column: Carbomix H-NP10: 8%, 7.8 × 300 mm, Sepax Technologies, Inc., Newark, DE, USA; detector: DAD, 214 nm, Agilent 1200 Series, Agilent Technologies Co., MNC, Santa Clara, CA, USA; eluent: 2.5 mmol/L H_2_SO_4_, 0.6 mL/min; temperature: 55 °C). Overnight ZA3 bacteria cultures of LAB grown in MRS broth were centrifuged at 8000 × *g* for 10 min and filtered through a 0.22 μm filter membrane. The organic acids, lactic acid, acetic acid, propionic acid, butyric acid, succinic acid, citric acid, and malic acid were detected.

### 4.12. Statistical Analyses

Each test was performed in triplicate. Data were analyzed by one-way analysis of variance (ANOVA) or paired t-test (SPSS 22.0). All data were shown as mean ± standard error of mean (SEM), and *p* < 0.05 indicated significant difference in statistics.

## 5. Conclusions

In this study, the inhibitory activities of 1100 LAB strains from different sources were tested to inhibit ETEC K88. Of which, ZA3, isolated from weaned pig feces, and identified as *L. plantarum* subsp. *plantarum,* had good inhibition ability and growth performance, excellent safety features, as well as good hydrophobicity and auto-aggregation, high survival rate in SGF and SIF, broad-spectrum activity against a wide range of microorganisms, and antibacterial substance (which may be attributed to organic acids). Therefore, ZA3 might be a suitable candidate for further study, due to its protective effects against ETEC K88 infections in weaned piglets.

## Figures and Tables

**Table 1:** Antimicrobial activities to Enterotoxigenic *Escherichia coli* (ETEC) K88 of representative lactic acid bacteria (LAB) isolates.

Isolates	Antimicrobial Activity	Separation Source	Isolates	Antimicrobial Activity	Separation Source
ZA1	+++	Weaned piglet feces	ZA21	+++	Rice silage
ZA2	++++		ZA22	+++	
ZA3	++++		ZA23	+++	
ZA4	+++		ZA24	++++	
ZA5	+++		ZA25	+++	
ZA6	+++		ZA26	+++	
ZA7	++++	Feed grass	ZA27	+++	
ZA8	++++		ZA28	++++	
ZA9	+++		ZA29	+++	Mixture silage
ZA10	++++		ZA30	++++	
ZA11	+++		ZA31	+++	
ZA12	+++		ZA32	+++	
ZA13	+++		ZA33	++++	
ZA14	+++		ZA34	++++	
ZA15	++++	Qula	ZA35	+++	
ZA16	+++		ZA36	+++	
ZA17	+++		ZA37	+++	Corn silage
ZA18	++++	Cassava silage	ZA38	+++	
ZA19	++++		ZA39	+++	
ZA20	+++		ZA40	+++	

Note: +++, diameter of inhibition zone: 18.00–22.00 mm; ++++, more than 22.00 mm; the diameter of inhibition zone including that of hole puncher (10.00 mm).

**Table 2:** Physiological and biochemical characteristics of selected LAB isolates.

Isolates	Growth at Temperature (°C)				Growth in NaCl (*w/v*, %)		Growth at pH									Fermentation Type
	5	10	45	50	3.0	6.5	3.0	3.5	4.0	4.5	5.0	5.5	6.0	8.0	9.0	
ZA1	w	+	+	+	+	+	w	+	+	+	+	+	+	+	+	Homo
ZA2	w	+	+	+	+	+	w	w	+	+	+	+	+	+	w	Homo
ZA3	+	+	+	w	+	+	+	+	+	+	+	+	+	+	+	Homo
ZA4	+	+	+	w	+	+	−	−	+	+	+	+	+	+	+	Homo
ZA5	w	w	+	w	+	+	−	w	+	+	+	+	+	+	+	Homo
ZA6	w	+	+	w	+	+	w	w	+	+	+	+	+	+	+	Hetero
ZA7	−	+	w	−	+	+	−	−	+	+	+	+	+	+	+	Homo
ZA8	w	+	+	w	+	w	+	+	+	+	+	+	+	+	+	Homo
ZA9	+	+	+	w	+	+	w	w	+	+	+	+	+	+	+	Homo
ZA10	w	+	w	−	+	+	+	+	+	+	+	+	+	+	+	Homo
ZA11	w	+	+	+	+	+	w	+	+	+	+	+	+	+	+	Homo
ZA12	+	+	+	+	+	+	−	+	+	+	+	+	+	+	+	Homo
ZA13	w	+	+	+	+	+	w	+	+	+	+	+	+	+	+	Homo
ZA14	w	+	+	+	+	w	w	+	+	+	+	+	+	+	+	Homo
ZA15	−	w	w	−	+	+	w	+	+	+	+	+	+	+	w	Hetero
ZA16	w	+	+	+	+	+	w	+	+	+	+	+	+	+	+	Homo
ZA17	−	+	w	+	+	w	−	w	w	+	+	+	+	+	+	Homo
ZA18	w	+	w	w	+	+	+	w	+	+	+	+	+	+	+	Hetero
ZA19	w	+	w	−	+	w	w	w	+	+	+	+	+	+	+	Homo
ZA20	w	+	+	+	+	w	w	w	+	+	+	+	+	+	+	Homo
ZA21	w	+	+	w	+	+	−	w	+	+	+	+	+	+	+	Homo
ZA22	−	+	+	+	+	w	−	−	w	+	+	+	+	+	+	Homo
ZA23	+	+	w	−	+	w	−	w	+	+	+	+	+	+	+	Homo
ZA24	+	+	w	w	+	w	−	w	w	+	+	+	+	+	+	Homo
ZA25	−	+	+	+	+	+	−	w	+	+	+	+	+	+	+	Homo
ZA26	−	+	w	w	+	+	w	+	+	+	+	+	+	+	+	Hetero
ZA27	w	+	+	+	+	w	−	−	w	+	+	+	+	+	+	Hetero
ZA28	+	+	+	w	+	+	w	+	+	+	+	+	+	+	+	Homo
ZA29	w	+	+	+	+	w	w	w	+	+	+	+	+	+	+	Homo
ZA30	−	+	+	w	+	+	−	−	w	+	+	+	+	+	+	Homo
ZA31	w	+	+	−	+	+	−	+	+	+	+	+	+	+	+	Hetero
ZA32	−	+	+	−	+	+	−	w	+	+	+	+	+	+	+	Hetero
ZA33	+	+	+	+	+	+	+	+	+	+	+	+	+	+	+	Hetero
ZA34	w	+	+	w	+	+	+	+	+	+	+	+	+	+	+	Homo
ZA35	w	+	w	−	+	w	−	w	+	+	+	+	+	+	+	Homo
ZA36	−	+	+	w	+	+	w	w	+	+	+	+	+	+	w	Homo
ZA37	w	+	+	−	+	+	−	−	+	+	+	+	+	+	+	Homo
ZA38	w	+	+	+	+	w	−	w	+	+	+	+	+	+	+	Homo
ZA39	−	+	+	+	+	+	−	w	+	+	+	+	+	+	+	Homo
ZA40	w	+	+	+	+	w	w	+	+	+	+	+	+	+	+	Homo

Note: +, positive; −, negative; w, weakly positive; homo, homofermentative; hetero, heterofermentative.

**Table 3:** Detection of virulence factor gene, biogenic amine production gene, and antibiotic resistance gene in ZA3.

Isolate	Virulence Factors Genes			Biogenic Amines Genes			Antibiotic Resistance Genes	
	ace	gelE	cylA	hdc	tdc	odc	vanA	tetM
ZA3	-	-	-	-	-	-	-	-

**Table 4:** Antibiotic susceptibility, MIC and cut-off value of isolate ZA3.

Item	CB	CZ	AM	GM	NOR	CC	P	E	C	AK
MIC (µg/mL)	0.5	10	1	64	>512	8	64	1	8	20
cut-off value (µg/mL)	2	2	2	16	4	2	2	8	8	64
ZA3	S	R	S	R	R	R	R	S	S	S

Notes: S: Susceptible; I: Intermediate resistant; R: Resistant. Refer to the latest Clinical and Laboratory Standards Institute (CLSI) standard. CB: carbenicillin; CZ: cefamezin; AM: ampicillin; GM: gentamicin; NOR: norfloxacin; CC: clindamycin; P: penicillin; E: erythromycin; C: chloramphenicol; AK: amikacin. Cut-off value cited from EFSA.

**Table 5:** Antibacterial spectrum of ZA3.

Isolate	Indicator Bacteria						
	*E. coli*	*P. aeruginosa*	*S. aureus*	*B. subtilis*	*L. monocytogenes*	*M. luteus*	*S. enterica*
ZA3	+++	++++	+++	+++	++++	+++	+++

Notes: 1. +++, diameter of inhibition zone: 18.00–22.00 mm; ++++, more than 22.00 mm; the diameter of inhibition zone including that of hole puncher (10.00 mm); 2. *E. coli*: *Escherichia coli* ATCC 11775^T^; *P. aeruginosa*: *Pseudomonas aeruginosa* ATCC 15692^T^; *S. aureus*: *Staphylococcus aureus* ATCC 6538^T^; *B. subtilis*: *Bacillus subtilis* ATCC 19217^T^; *L. monocytogenes*: *Listeria monocytogenes* ATCC 51719^T^; *M. luteus*: *Micrococcus luteus* ATCC 4698^T^; *S. enterica*: *Salmonella enterica* ATCC 43971^T^.

**Table 6:** Carbohydrate utilization patterns of ZA3.

Substrate	ZA3	Substrate	ZA3
Glycerol	−	Salicin	−
D-Arabinose	−	Cellobiose	−
L-Arabinose	−	Maltose	+
Ribose	w	Lactose	+
D-Xylose	−	Melibiose	+
Galactose	+	Saccharose	+
D-Glucose	+	Trehalose	+
D-Fructose	+	Melezitose	−
Mannose	+	D-Raffinose	−
L-Sorbose	+	Starch	−
Rhamnose	+	Xylitol	−
Dulcitol	−	L-Arabitol	−
Inositol	−	Gluconate	+
Mannitol	+	N-acetyl glucosamine	+
Sorbitol	+	Amygdalin	+
Glycerol	−	Salicin	−
D-Arabinose	−	Cellobiose	−
L-Arabinose	−	Maltose	+
Ribose	w	Lactose	+

Note: +, positive; −, negative; w, weakly positive.

**Table 7:** Antimicrobial activity of ZA3 to ETEC K88 after different treatment.

Treatment	Antimicrobial Activity
ZA3	
fermentation liquid	+++
supernatant	+++
hydrogen peroxide	+++
proteinase K	++
pepsinum	+++
tryptase	+++
pH	
3.0	+++
4.0	+++
4.5	++
5.0	+
5.5	−
6.0	−
6.5	−
7.0	−
10.0	−

Note: +, diameter of inhibition zone: 10.00–14.00 mm; ++, 14.0–18.00 mm; +++, 18.0–22.00 mm; −, no inhibition zone was detected; the diameter of inhibition zone including that of hole puncher (10.00 mm).

**Table 8:** Information of bacterial strains in this article.

Strain	Source	Strain	Source
ETEC K88	CVCC	*Salmonella enterica* ATCC 43971^T^	CGMCC
*Staphylococcus aureus* ATCC 6538^T^	Store in laboratory	*B. subtilis* ATCC 19217^T^	Store in laboratory
*Escherichia coli* ATCC 11775^T^	Store in laboratory	*Listeria monocytogenes* ATCC 51719^T^	Store in laboratory
*P. aeruginosa* ATCC 15692^T^	Store in laboratory	*M. luteus* ATCC 4698^T^	Store in laboratory
*Enterococcus faecalis* ATCC 29212^T^	CICC		

Notes: 1. CVCC, China Veterinary Culture Collection Center; CGMCC, China General Microbiological Culture Collection Center; CICC, China Center of Industrial Culture Collection; 2. *B. subtilis*: *Bacillus subtilis* ATCC 19217^T^; *P. aeruginosa*: *Pseudomonas aeruginosa* ATCC 15692^T^; *M. luteus*: *Micrococcus luteus* ATCC 4698^T^; *E. faecalis*: *faecalis* ATCC 29212^T^.

## Data Availability

The 16Sr RNA gene sequence of strains ZA3, ZA2, ZA28, ZA8, ZA10, ZA19, ZA33, ZA18, and ZA34 used to support the findings of this study have been deposited in the GenBank repository with accession number MT597900, MT597901, MT597902, MT597903, MT597904, MT597905, MT597906, MF597907, and MF597910, respectively.
